# Comparison of epidermal growth factor receptor tyrosine kinase inhibitors for patients with lung adenocarcinoma harboring different epidermal growth factor receptor mutation types

**DOI:** 10.1186/s12885-020-07765-6

**Published:** 2021-01-11

**Authors:** Sojung Park, Sung Yong Lee, Dojin Kim, Yun Su Sim, Jeong-Seon Ryu, Juwhan Choi, Su Hwan Lee, Yon Ju Ryu, Jin Hwa Lee, Jung Hyun Chang

**Affiliations:** 1 Division of Pulmonary and Critical Care Medicine, College of Medicine, Ewha Womans University, 1071 Anyangcheon-Ro, Yangcheon-gu, Seoul, 07985 Republic of Korea; 2 Division of Respiratory, Allergy and Critical Care Medicine, Department of Internal Medicine, Korea University Guro Hospital, Seoul, Republic of Korea; 3 Division of Allergy and Respiratory Medicine, Soonchunhyang University Bucheon Hospital, Bucheon, Gyeonggi Republic of Korea; 4 Division of Pulmonary, Allergy and Critical Care Medicine, Department of Internal Medicine, Hallym University Kangnam Sacred Heart Hospital, Seoul, Republic of Korea; 5 Department of Internal Medicine, Inha University College of Medicine, Incheon, Republic of Korea

**Keywords:** Epidermal growth factor receptor, Non-small cell lung cancer, Adenocarcinoma, Survival, Tyrosine kinase inhibitor

## Abstract

**Background:**

Epidermal growth factor receptor (EGFR) mutations in non–small-cell lung cancer predict sensitivity to EGFR tyrosine kinase inhibitors (TKIs). *EGFR* mutation types are associated with efficacy of EGFR TKIs. We investigated the clinical outcomes of afatinib, erlotinib, and gefitinib according to *EGFR* mutation type in patients with lung adenocarcinoma.

**Methods:**

Between May 2010 and December 2018, we investigated 363 patients with advanced lung adenocarcinoma harboring *EGFR* mutations who received EGFR TKIs. Efficacies of EGFR TKIs such as response rate, progression-free survival (PFS), and overall survival (OS) were retrospectively evaluated according to exon 19 deletion (E19del), L858R point mutation (L858R) and uncommon mutations.

**Results:**

The frequency of E19del was 48.2%, that of L858R was 42.4%, and that of uncommon mutations was 9.4%. E19del and L858R were associated with superior PFS and OS compared with uncommon mutations. Erlotinib showed significantly inferior OS than other TKIs (30.8 ± 3.3 in erlotinib vs. 39.1 ± 4.3 in afatinib vs. 48.4 ± 6.3 in gefitinib; *p* = 0.031) in patients with L858R. Gefitinib showed significantly inferior PFS (4.6 ± 1.1 in gefitinib vs. 11.6 ± 2.7 in afatinib vs. 10.6 ± 2.7 in erlotinib; *p =* 0.049) in patients with uncommon mutations.

**Conclusion:**

Afatinib was significantly associated with a longer PFS, presenting constant effectiveness in all *EGFR* mutation types. Caution may be needed on the use of erlotinib for L858R and the use of gefitinib for uncommon *EGFR* mutations.

**Supplementary Information:**

The online version contains supplementary material available at 10.1186/s12885-020-07765-6.

## Background

Somatic mutations in the epidermal growth factor receptor (EGFR) gene have been associated with sensitivity to EGFR tyrosine kinase inhibitors (TKIs) in non-small-cell lung cancer (NSCLC) [1–4]. *EGFR* mutations occur mainly in exons 18 through 21, which encompass most of the tyrosine kinase binding domain of EGFR. Previous studies demonstrated that exon 19 deletions (E19del) and L858R point mutation (L858R) are associated with better outcomes than uncommon *EGFR* mutations, such as G719X in exon 18 and exon 20 insertion [5–7]. For small case numbers and the highly heterogeneous spectrum of uncommon mutations, the efficacy of EGFR TKIs in patients with uncommon *EGFR* mutations has not been fully elucidated.

Afatinib, erlotinib, and gefitinib have been approved based on randomized trials showing superior progression-free survival (PFS), objective response rate (ORR), and more favorable safety profiles when compared with platinum-based chemotherapy in patients with EGFR-mutant NSCLC [1–4]. There are different mechanisms of action between the first-generation EGFR TKIs, which reversibly bind to and inhibit EGFR signaling, and the second-generation afatinib, which irreversibly blocks signaling from all relevant homo-dimers and hetero-dimers of the ErbB family of receptors [8]. However, there are no guidelines for the most appropriate choice of first-line treatment for a given patient because no prospective head-to-head comparison study has been conducted.

The aim of the present study was to investigate and compare the treatment efficacy of afatinib, erlotinib, and gefitinib according to *EGFR* mutation type in patients with lung adenocarcinoma.

## Methods

### Study population

We screened patients with advanced lung adenocarcinoma that harboured *EGFR* mutations from five secondary or tertiary medical institutes between May 2010 and December 2018. Of these, patients who received EGFR TKIs for at least 1 month and underwent disease status re-evaluation were included in the present study. Patients for whom treatment was discontinued for reasons other than disease progression, such as poor performance status, infection, transfer to another hospital, and dropout, were excluded. Demographic and clinicopathological information of patients were retrospectively reviewed through electronic medical records. The following variables were assessed: age, sex, body mass index (BMI), smoking history, *EGFR* mutation type, stage according to 8th TNM classification, treatment regimen, treatment response, PFS, overall survival (OS), and drug adverse events (AEs).

*EGFR* mutation types were classified as follows: E19del, L858R, and uncommon mutations that are defined as mutations other than E19del or L858R. Uncommon mutations also included concomitant mutation in two or more exons, therefore, concomitant mutation in E19del or L858R and in another exon was regarded as an uncommon mutation.

Informed consent was waived because of the retrospective study design, and the study was approved by the institutional review boards of all participating institutes. (Ewha Womans University: EUMC 2020–07–010-002, Korean University: 2020GR0347, Soonchunhyang University: SCHBC2020–08-027, Hallym University: 2020–07-014, and Inha University: 2020–07-008).

### Evaluation of efficacy

Each drug was orally administered once a day until progressive disease (PD) or unacceptable toxicity was noted. All patients were recommended to visit the clinic within 2 weeks after starting TKIs. If serious AEs were not noted at the first visit, patients visited the clinic every 4 or 8 weeks thereafter. Chest computed tomography (CT) was performed every 1 to 3 months to evaluate treatment response. ORR was calculated by checking the best response based on the Response Evaluation Criteria in Solid Tumors version 1.1 [9]. PFS was defined as the time from EGFR TKI commencement to either documented disease progression or death from any cause. OS was defined as the time from diagnosis to death. Drug AEs were graded according to the National Cancer Institute Common Terminology Criteria for Adverse Events (CTCAE) version 4.0 [10]. The date of data cutoff was December 31, 2019.

### Histology and molecular testing

Tumor tissues were obtained by CT-guided needle biopsy, bronchoscopic biopsy, and surgical resection. DNA was extracted from archived paraffin-embedded tumor tissues. *EGFR* mutation testing was conducted in two ways according to the facilities and timing of each institution. Between 2010 and 2013, *EGFR* mutation status was analyzed by ISU ABXIS Co Ltd. (Seoul, Korea), an independent commercial laboratory. From 2013 to 2016, PNAClamp™ EGFR Mutation Detection Kit with the PNA-mediated PCR clamping method (Panagene, Daejeon, Korea) was used at each institute to identify *EGFR* mutations in accordance with the manufacturer’s instructions. The subtypes of detected mutations are described in Additional file 1.

### Statistical analysis

Either Pearson chi-square test or Fisher’s exact test was used to compare categorical variables, and a one-way analysis of variance test was used to compare continuous variables. PFS and OS curves were plotted using the Kaplan-Meier method and were compared using the log-rank test. Variables selected by univariate analysis (*p* < 0.1) were evaluated in a multivariate analysis using the Cox proportional hazard model. All tests of significance were two-sided, and differences among groups were considered significant when the *p*-value was < 0.05. All statistical analyses were performed with SPSS software version 22.0 (IBM Corporation, Armonk, NY, USA).

## Results

### Patient characteristics-

**Fig. 1:**
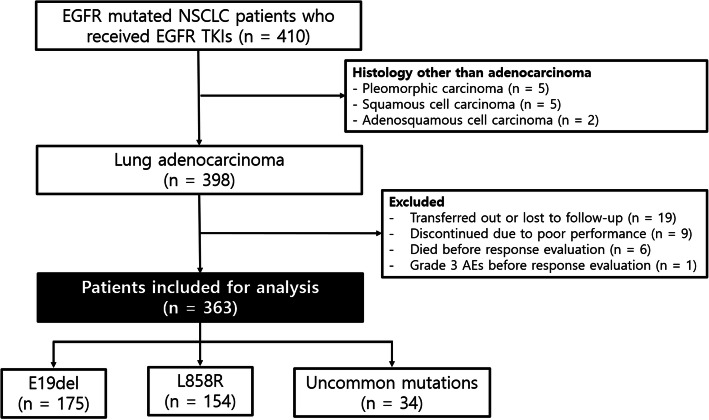
Inclusion and exclusion criteria of patient enrolment. EGFR, epidermal growth factor receptor; NSCLC, non-small-cell lung cancer; TKIs, tyrosine kinase inhibitors; AEs, adverse events; E19del, exon 19 deletion; L858R, L858R point mutation; uncommon, uncommon mutations

From May 2010 to December 2018, a total of 410 patients with advanced NSCLC underwent mutation testing for *EGFR* and received EGFR TKIs. Excluding 47 patients who met the exclusion criteria, 363 patients with advanced lung adenocarcinoma were finally included in the present study (Fig. 1). The mean age was 67.6 ± 11.0 years; 62.3% were females (Table 1). The frequency of E19del was 48.2%, that of L858R was 42.4%, and that of uncommon mutations was 9.4%. Of the 363 patients, 292 (80.4%) received EGFR TKIs as first-line treatment, and 10 (2.8%) received them as third-line treatment or beyond. No significant difference was found in treatment regimen among patients with each *EGFR* mutation type; afatinib was given to 102 (28.1%) patients, erlotinib to 139 (38.3%), and gefitinib to 122 (33.6%, *p* = 0.422).

**Table 1:** Baseline characteristics of the study population

	Total (*n* = 363)	E19del (*n* = 175)	L858R (*n* = 154)	Uncommon (*n* = 34)	*p* value
Age, mean ± SD	67.6 ± 11.0	66.5 ± 11.8	69.1 ± 9.9	66.1 ± 10.8	0.076
Sex, male	137 (37.7)	68 (38.9)	59 (38.3)	10 (29.4)	0.572
BMI, kg/m^2^	24.0 ± 3.5	24.2 ± 3.6	23.6 ± 3.3	25.5 ± 4.0	0.012
Smoking status					0.691
Never smoker	258 (71.3)	123 (70.3)	109 (71.2)	26 (76.5)	
Ex-smoker	76 (21.0)	36 (20.6)	35 (22.9)	5 (14.7)	
Current smoker	28 (7.7)	16 (9.1)	9 (5.9)	3 (8.8)	
Stage					0.361
Stage III	31 (8.5)	15 (8.6)	11 (7.1)	5 (14.7)	
Stage IV	332 (91.5)	160 (91.4)	143 (92.9)	29 (85.3)	
Previous chemotherapy					0.442
0	292 (80.4)	137 (78.3)	126 (81.8)	29 (85.3)	
1	61 (16.8)	34 (19.4)	24 (15.6)	3 (8.8)	
≥2	10 (2.8)	4 (2.3)	4 (2.6)	2 (5.9)	
EGFR TKIs					0.422
Afatinib	102 (28.1)	53 (30.3)	38 (24.7)	11 (32.4)	
Erlotinib	139 (38.3)	69 (39.4)	61 (39.6)	9 (26.5)	
Gefitinib	122 (33.6)	53 (30.3)	55 (35.7)	14 (41.2)	

Data are shown as n (%) per each group, unless otherwise noted

*E19del* exon 19 deletion, *L858R* L858R point mutation, *Uncommon* uncommon mutations, *SD* standard deviation, *BMI* body mass index, *EGFR* epidermal growth factor receptor, *TKI* tyrosine kinase inhibitor

### Objective response rate

During a median follow-up duration of 24.9 months (range, 1.2–110.0 months), 209 patients (57.6%) died and 63 (17.4%) were still using EGFR TKIs at the time of data cutoff. The overall ORR and disease control rate (DCR) for EGFR TKIs was 73.3 and 93.1%, respectively (Table 2). ORRs were 76.0, 76.0, and 47.1% for E19del, L858R, and uncommon mutations, respectively (*p* < 0.001). However, no significant differences were found in ORR according to EGFR TKI regimen. In patients harboring E19del and L858R, no significant difference was found in ORR (Table 3). In patients harboring uncommon mutations, DCRs were 81.8, 88.9, and 57.1% in afatinib-, erlotinib-, and gefitinib-treated patients, although they did not reach statistical significance thresholds (*p* = 0.182).

**Table 2:** Response rate of epidermal growth factor receptor tyrosine kinase inhibitors according to mutation type and treatment regimen

	CR	PR	SD	PD	*p* value
Total (*n* = 363)	1 (0.3)	265 (73.0)	72 (19.8)	25 (6.9)	< 0.001
EGFR mutation types					
E19del (*n* = 175)	1 (0.6)	132 (75.4)	33 (18.9)	9 (5.1)	
L858R (*n* = 154)	0	117 (76.0)	30 (19.5)	7 (4.5)	
Uncommon (*n* = 34)	0	16 (47.1)	9 (26.5)	9 (26.5)	
EGFR TKIs					0.914
Afatinib (*n* = 102)	0	74 (72.5)	21 (20.6)	7 (6.9)	
Erlotinib (*n* = 139)	1 (0.7)	100 (71.9)	29 (20.9)	9 (6.5)	
Gefitinib (*n* = 122)	0	91 (74.6)	22 (18.0)	9 (7.4)	

Data are shown as n (%) per each group

*CR* complete response, *PR* partial response, *SD* stable disease, *PD* progressive disease, *E19del* exon 19 deletion, *L858R* L858R point mutation, *Uncommon* uncommon mutations, *EGFR* epidermal growth factor receptor, *TKIs* tyrosine kinase inhibitors

**Table 3:** Response rate of epidermal growth factor receptor tyrosine kinase inhibitors according to mutation type

	CR	PR	SD	PD	*p* value
Total (*n* = 363)	1 (0.3)	265 (73.0)	72 (19.8)	25 (6.9)	
E19del (*n* = 175)					0.785
Afatinib (*n* = 53)	0	38 (71.7)	12 (22.6)	3 (5.7)	
Erlotinib (*n* = 69)	1 (1.4)	54 (78.3)	10 (14.5)	4 (5.8)	
Gefitinib (*n* = 53)	0	40 (75.5)	11 (20.8)	2 (3.8)	
L858R (*n* = 154)					0.471
Afatinib (*n* = 38)	0	30 (78.9)	6 (15.8)	2 (5.3)	
Erlotinib (*n* = 61)	0	42 (68.9)	15 (24.6)	4 (6.6)	
Gefitinib (*n* = 55)	0	45 (81.8)	9 (16.4)	1 (1.8)	
Uncommon (*n* = 34)					0.332
Afatinib (*n* = 11)	0	6 (54.5)	3 (27.3)	2 (18.2)	
Erlotinib (*n* = 9)	0	4 (44.4)	4 (44.4)	1 (11..1)	
Gefitinib (*n* = 14)	0	6 (42.9)	2 (14.3)	6 (42.9)	

Data are shown as n (%) per each group

*CR* complete response, *PR* partial response, *SD* stable disease, *PD* progressive disease, *E19del* exon 19 deletion, *L858R* L858R point mutation, *Uncommon* uncommon mutations

### Survival outcomes

**Fig. 2:**
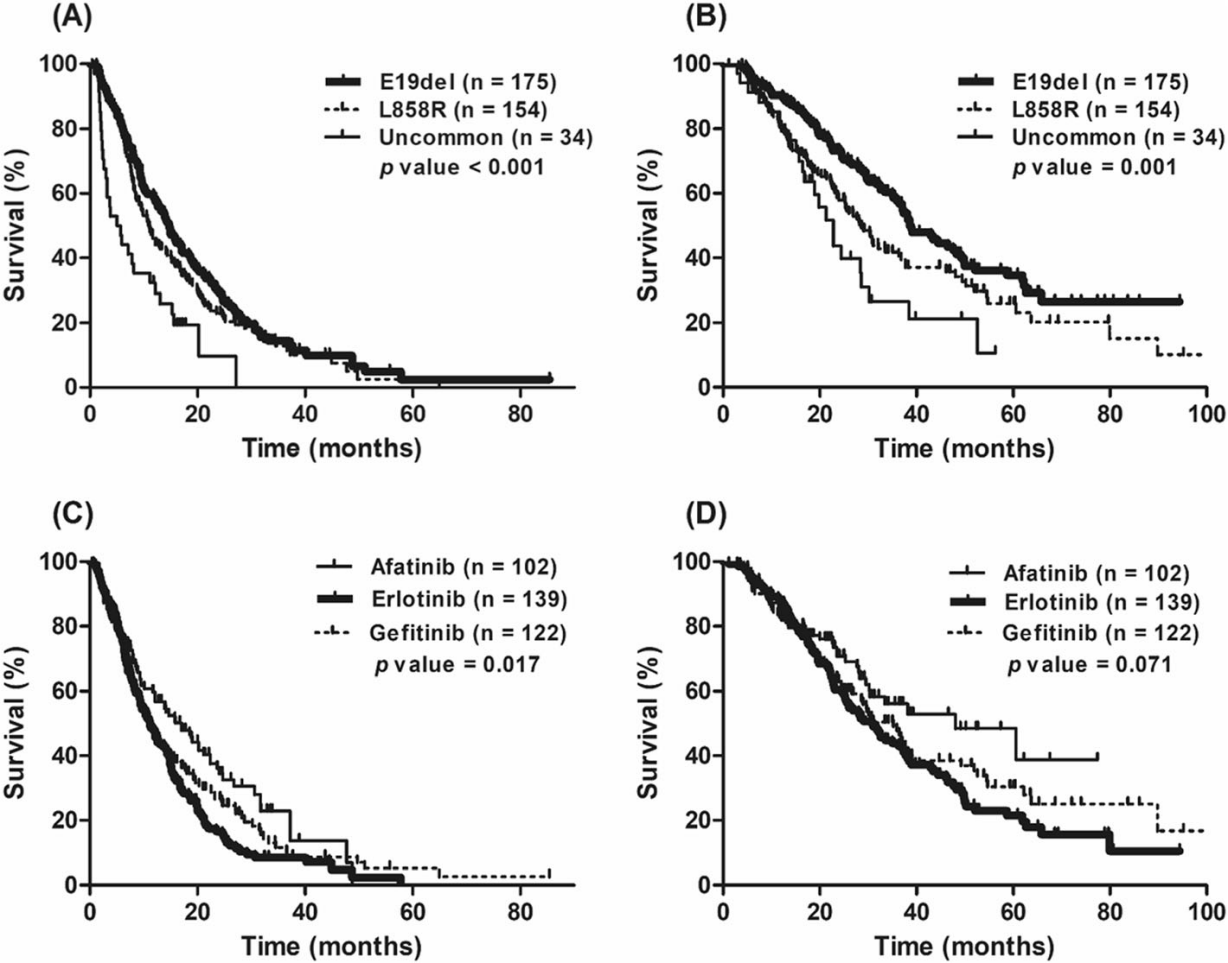
Survival curves in lung adenocarcinoma patients harbouring *EGFR* mutations who received tyrosine kinase inhibitors. **a** Comparison of progression-free survival according to mutation type. **b** Comparison of overall survival according to mutation type. **c** Comparison of progression-free survival according to treatment regimen. **d** Comparison of overall survival according to treatment regimen

The median PFS of all patients was 12.1 months (95% confidence interval [CI], 10.2–14.0). A significant difference was found in the median PFS among E19del, L858R, and uncommon mutations: 14.7 months (95% CI, 12.6–16.8) for E19del, 10.9 months (95% CI, 9.0–12.7) for L858R, and 5.0 months (95% CI, 1.8–14.0) for uncommon mutations (*p* < 0.001; Fig. 2a). The median OS of all patients was 33.4 months (95% CI, 29.0–37.8) and a significant difference was found in the median OS among *EGFR* mutation types: 38.6 months (95% CI, 32.9–44.3) for E19del, 28.6 months (95% CI, 24.3–32.9) for L858R, and 22.8 months (95% CI, 18.2–27.4) for uncommon mutations (*p* = 0.001; Fig. 2b).

The median PFS times for afatinib, erlotinib, and gefitinib were 17.0 months (95% CI, 11.6–22.4), 11.2 months (95% CI, 8.9–13.5), and 10.9 months (95% CI, 8.3–13.5; *p* = 0.017; Fig. 2c). However, no significant difference was found in OS according to EGFR TKI regimen (Fig. 2d).

**Fig. 3:**
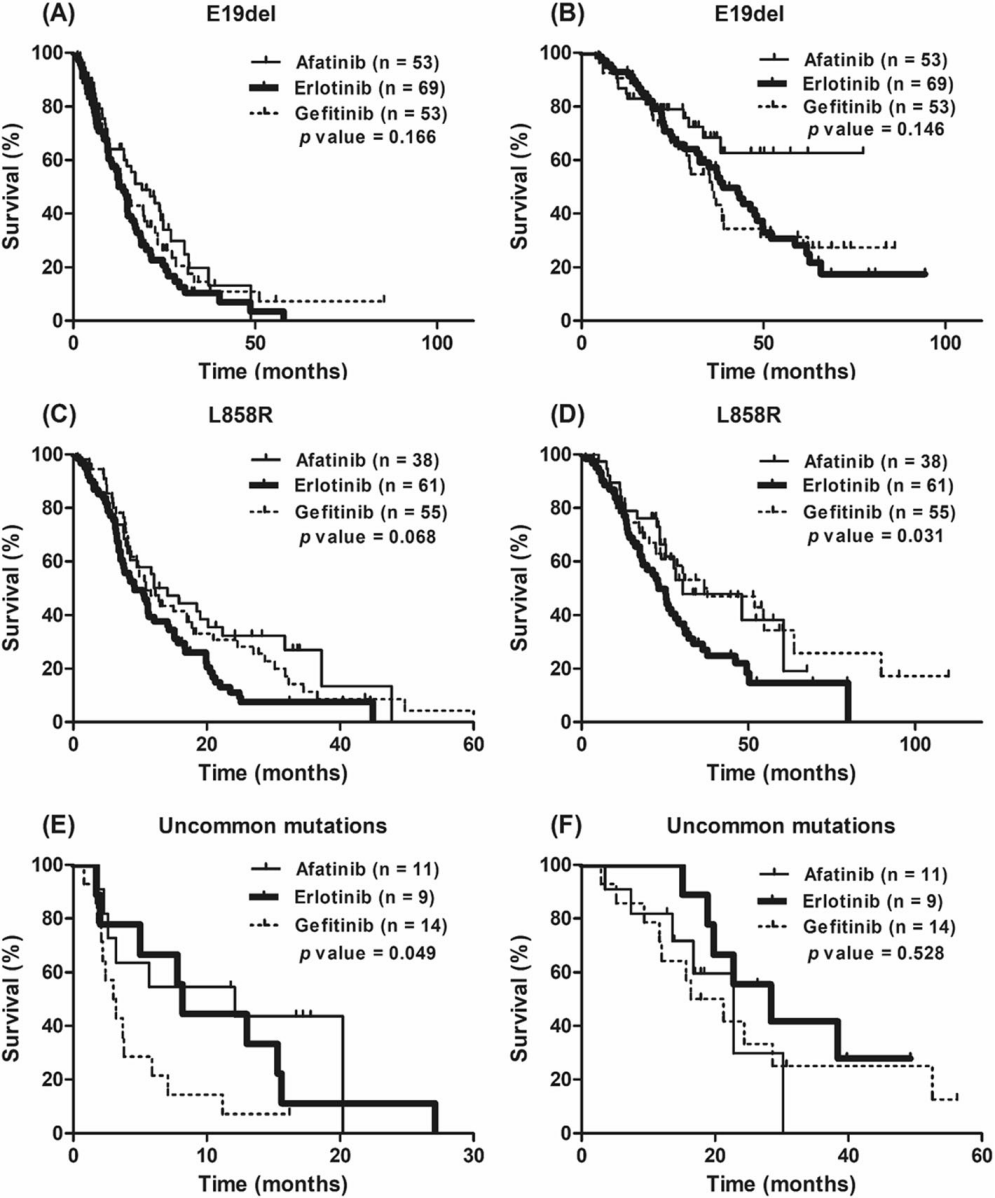
Survival curves in lung adenocarcinoma patients harbouring *EGFR* mutations according to each mutation type. **a**, **b** Comparison of progression-free survival and overall survival for exon 19 deletion. **c**, **d** Comparison of progression-free survival and overall survival for L858R point mutation. **e**, **f** Comparison of progression-free survival and overall survival for uncommon mutations

In patients harboring E19del, no significant differences were found in PFS and OS according to EGFR TKI regimen (Fig. 3a and b). In patients harboring L858R mutation, the median PFS times for afatinib, erlotinib, and gefitinib were 12.1 months (95% CI, 6.1–18.1), 9.2 months (95% CI, 5.6–12.7), and 10.9 months (95% CI, 7.8–13.9), respectively (*p* = 0.068; Fig. 3c). A significant difference was found in OS according to EGFR TKI regimen: 30.3 months (95% CI, 10.0–50.6) in afatinib-, 23.1 months (95% CI, 18.2–28.0) in erlotinib-, and 36.8 months (95% CI, 12.3–61.3) in gefitinib-treated patients (*p* = 0.031; Fig. 3d). In patients harboring uncommon mutations, a significant difference was found in the median PFS time according to EGFR TKI regimen: 12.1 months (95% CI, 0.0–25.6) in afatinib-, 8.2 months (95% CI, 7.0–9.5) in erlotinib-, and 3.0 months (95% CI, 1.4–4.5) in gefitinib-treated patients (*p* = 0.049, Fig. 3e). No significant difference was found in OS according to EGFR TKI regimen in patients harboring uncommon mutations (Fig. 3f).

Univariate analysis revealed that BMI, smoking status, *EGFR* mutation type, type of EGFR TKI, and line of treatment were associated with PFS. In multivariate analysis, BMI (hazard ratio [HR], 0.963; 95% CI, 0.932–0.994; *p* = 0.021) and *EGFR* mutation type (HR, 2.806; 95% CI, 1.850–4.255; *p* < 0.001) were associated with PFS (Additional file 2).

We evaluated isolated central nervous system (CNS) failure and bone failure without systemic disease progression during EGFR TKI treatment. No significant difference was found in the rate of isolated CNS failure among treatment regimen; however, patients who received erlotinib experienced significantly more isolated bone failure compared with those who received other regimens (18.7% in erlotinib versus 9.8% in afatinib and 9.0% in gefitinib, respectively, *p* = 0.036).

### Serious adverse events

We evaluated the incidence of serious AEs which were defined as CTCAE grades 3 to 5. The overall incidence of serious AEs was 7.4% (Additional file 3). The most common AEs were skin rash (5.0%), followed by gastrointestinal toxicity (2.2%) and myalgia (0.3%). The incidence of serious AEs was comparable among the three treatment groups. No treatment-related deaths occurred for all three drugs.

### Acquired mutation after use of EGFR TKIs

Among 300 patients who discontinued EGFR TKIs at the time of data cutoff, 101 patients (33.7%) underwent re-biopsy for investigation of acquired resistance mutations. As a result, 50 cases (49.5%) acquired T790M mutation and one case transformed to small-cell lung cancer. Three cases (4.0%) were converted to *EGFR* wild-type (Table 4). No difference was found in the rate of acquired resistance mutations according to initial *EGFR* mutation type and treatment regimen.

**Table 4:** Acquired resistance mechanism after the use of epidermal growth factor receptor tyrosine kinase inhibitors according to mutation type and treatment regimen

	No change	T790M	SCLC	Wild-type	*p* value
Total (*n* = 101)	46 (45.5)	50 (49.5)	1 (1.0)	4 (4.0)	
EGFR mutation types					
E19del (*n* = 51)	21 (41.2)	27 (52.9)	0	3 (5.9)	0.447
L858R (*n* = 42)	19 (45.2)	21 (50.0)	1 (2.4)	1 (2.4)	0.601
Uncommon (*n* = 8)	6 (75.0)	2 (25.0)	0	0	0.371
EGFR TKIs					
Afatinib (*n* = 38)	21 (55.3)	14 (36.8)	0	3 (7.9)	0.130
Erlotinib (*n* = 32)	12 (37.5)	19 (59.4)	1 (3.1)	0	0.168
Gefitinib (*n* = 31)	13 (41.9)	17 (54.8)	0	1 (3.2)	0.747

A total of 101 patients underwent re-biopsy for investigation of acquired resistance after discontinuation of EGFR TKI. The most common mechanism was acquisition of T790M mutation, followed by conversion to *EGFR* wild-type, and transformation to small-cell lung cancer. No difference was found in the rate of acquired resistance mutations according to initial *EGFR* mutation type and treatment regimen

Data are shown as n (%) per each group

*SCLC* small-cell lung cancer, *E19del* exon 19 deletion, *L858R* L858R point mutation, *Uncommon* uncommon mutations

## Discussion

Afatinib, erlotinib, and gefitinib are recommended for the first-line treatment of NSCLC harboring *EGFR* mutations. Although these three drugs have all been used, the optimal choice for each patient is uncertain, as comparative studies have been scarce. The present study demonstrated that significantly different OS were found among EGFR TKIs in patients with L858R, as well as varying PFS in those with uncommon *EGFR* mutations, although multivariate analysis showed that a certain TKI regimen was not associated with superior PFS in a particular *EGFR* mutation type.

The WJOG 5108 L study which compared gefitinib with erlotinib demonstrated that no significant difference was found in PFS between the two TKIs [11]. The LUX-Lung 7 trial which compared afatinib with gefitinib demonstrated that afatinib showed a superior PFS compared with gefitinib [12]. However, this survival gain was not identified when it was analyzed separately for E19del and L858R. The present study showed that patients with L858R who received erlotinib had a tendency to experience a shortened PFS compared with those who received other regimens, and patients with uncommon mutations who received gefitinib had a shorter PFS compared with those who received other regimens. However, afatinib showed consistent effectiveness for all *EGFR* mutation types without a significantly increased risk of serious AEs. We assumed that this constant effectiveness might have caused superior PFS of afatinib in the total study population but not in a certain *EGFR* mutation type. The WJOG 5108 L study showed that the HR was in favor of gefitinib for L858R mutation, and erlotinib for uncommon mutations, although they did not reach statistical significance. Additionally, Kim et al. showed a similar tendency for PFS, although they also could not reach the statistical threshold [13]. Therefore, a large, randomized prospective study is needed to confirm that there are differences in the treatment efficacy of the three EGFR TKIs according to *EGFR* mutation type.

The incidence of uncommon mutations was 9.4% in the present study. The low prevalence and high heterogeneity make the comprehensive evaluation of the treatment effectiveness of EGFR TKIs for uncommon *EGFR* mutations difficult. *EGFR* exon 20 insertions and de novo T790M mutations are considered resistance mutations [14–16]. However, G719X, S768I, and L861Q may be sensitizing mutations [14, 17–19]. A combined post-hoc analysis of the LUX-Lung 2, 3, and 6 trials demonstrated that afatinib showed a median PFS of 10.7 months for point mutations and duplications in exons 18–21, 2.9 months for de novo T790M mutations, and 2.7 months for exon 20 insertions [18]. Tu et al. demonstrated that patients with compound L858R or G719X who received first-generation EGFR TKIs showed a comparable PFS with classical *EGFR* mutations, whereas those with T790M mutation or exon 20 insertion showed median PFS rates of 1.0 and 3.0 months, respectively [20]. Previous studies could not show a definite survival benefit of afatinib but a tendency for improved PFS and ORR compared with first-generation EGFR TKIs [21, 22]. We found that afatinib showed comparable efficacy with erlotinib in uncommon mutations. Therefore, afatinib or erlotinib may be recommended for first-line treatment in patients harboring uncommon *EGFR* mutations.

The incidence of serious AEs was lower than in previous studies [3, 4, 11]. This finding could be related to the limitation of retrospective studies, which might have omitted such cases. However, we took serious AEs that required medical intervention into account without mild AEs, and thus the possibility of omitting serious cases might be small. We assumed that appropriate dose reduction with or without temporary interruption, best supportive care, and racial differences might cause a low incidence of serious AEs.

The present study has several limitations. First, it is a retrospective observational study. The choice of EGFR TKI was based on the physician’s clinical judgment or preference, which might result in a selection bias. Second, the uncommon mutation cohort was relatively small and highly heterogeneous, which made subgroup analyses difficult. Additionally, the large number of patients with E19del subtype was unspecified and the minor type of E19del might be misclassified to E19del [23]. Third, only 33.7% of patients underwent re-biopsy to examine the resistance mechanism, which might cause selection bias and reduce the reliability of the result for the acquired resistance mechanism according to EGFR TKI or *EGFR* mutation type. Additionally, next-generation sequencing was not routinely performed, so there might be missed resistance mechanisms such as *MET* amplification and *BRAF* mutation. Further experimental approach to investigate the resistance mechanism according to *EGFR* mutation type and treatment regimen is needed. Lastly, National Comprehensive Cancer Network guideline recommends osimertinib as a preferred first line regimen in patients with *EGFR*-mutant NSCLC since version 1.2019 [24]. The present study was conducted with patients who had received EGFR TKI during May 2010 and December 2018, therefore, no patients had received osimertinib as a first line treatment. In addition, osimertinib is not yet covered by national insurance as first line treatment in Korea and many *EGFR*-mutant patients cannot afford the expense. Therefore, afatinib, erlotinib, and gefitinib still need to be selected as a first-line therapy in many cases. Indeed, 37 patients received osimertinib after acquiring T790M mutation.

## Conclusions

Afatinib was significantly associated with a longer PFS, presenting constant effectiveness in all *EGFR* mutation types, although multivariate analysis showed that a specific TKI regimen was not associated with superior PFS in a particular *EGFR* mutation type. However, caution may be needed on the use of erlotinib for L858R and the use of gefitinib for uncommon *EGFR* mutations.

## Supplementary Information

**Additional file 1 Table S1.** *EGFR* mutation subtypes.

**Additional file 2 Table S2.** Univariate and multivariate Cox proportional hazard analysis for progression-free survival in patients harboring epidermal growth factor receptor mutation and received tyrosine kinase inhibitors.

**Additional file 3 Table S3.** Drug-related serious adverse events.

## Data Availability

The datasets used and/or analysed during the current study available from the corresponding author on reasonable request.

## Abbreviations

EGFR: Epidermal growth factor receptor
TKIs: Tyrosine kinase inhibitors
NSCLC: Non-small-cell lung cancer
E19del: Exon 19 deletions
L858R: L858R point mutation
PFS: Progression-free survival
ORR: Objective response rate
BMI: Body mass index
OS: Overall survival
AEs: Adverse events
PD: Progressive disease
CT: Computed tomography
CTCAE: Common Terminology Criteria for Adverse Events
DCR: Disease control rate
CI: Confidence interval
HR: Hazard ratio
CNS: Central nervous system
