# Supplementary material for: Comparison of epidermal growth factor receptor tyrosine kinase inhibitors for patients with lung adenocarcinoma harboring different epidermal growth factor receptor mutation types
Source: BMC Cancer. 2021 Jan 11;21:52. doi: 10.1186/s12885-020-07765-6 (PMC7802134; doi:10.1186/s12885-020-07765-6)
Supplement: Supplementary file 1 — Additional file 1 Table S1. EGFR mutation subtypes. [file 12885_2020_7765_MOESM1_ESM.docx]

Supplementary Table S1. *EGFR* mutation subtypes

| Exon | type | Amino acid change | Base change | No^1^ | No of subtype^2^ | |
| --- | --- | --- | --- | --- | --- | --- |
| 18 | G719X unspecific |  |  | 9 | | 9 |
|  | Deletion |  | c.2127 2129delAAC | 1 | | 1 |
| 19 | E19del | p.E746_A750del | c.2235_2249 del 15 | 175 | | 6 |
|  |  | p.E746_A750del | c.2236_2250 del 15 |  |  | 6 |
|  |  | p.L747_T751del | c.2239_2253 del 15 |  |  | 1 |
|  |  | p.L747_A750>P | c.2239_2248 TTAAGAGAAG>C |  |  | 1 |
|  |  | p.L747_753>S | c.2240_2257 del 18 |  |  | 1 |
|  |  | p.L747_T751del | c.2240_2254 del 15 |  |  | 2 |
|  |  | 19 del unspecific |  |  |  | 158 |
| 20 | S768I | p.V769_D770insASV | c.2307_2308 ins9 | 1 | | 1 |
|  | E20 Ins.3dup | p.G773_V774insH | c.2319_2320 insCAC | 10 | | 4 |
|  | E20 Ins. unspecific |  |  |  |  | 6 |
|  | Deletion |  |  | 1 | | 1 |
| 21 | L858R | p.L858R | c.2573T>G | 156 | | 154 |
|  | L861Q | p.L861Q | c.2582T>A |  |  | 2 |
| Compound mutation | E19del +L858R |  |  | 10 | | 1 |
|  | G719X+S768I |  |  |  |  | 1 |
|  | E19del+S768I |  |  |  |  | 1 |
|  | G719X+L858R |  |  |  |  | 1 |
|  | L858R+T790M |  |  |  |  | 4 |
|  | E19del+T790M |  |  |  |  | 1 |
|  | G719X+L861Q |  |  |  |  | 1 |

**^1^**number of exon mutation type, **^2^**number of specific identified amino acid change
