# Supplementary material for: Comparison of epidermal growth factor receptor tyrosine kinase inhibitors for patients with lung adenocarcinoma harboring different epidermal growth factor receptor mutation types
Source: BMC Cancer. 2021 Jan 11;21:52. doi: 10.1186/s12885-020-07765-6 (PMC7802134; doi:10.1186/s12885-020-07765-6)
Supplement: Supplementary file 2 — Additional file 2 Table S2. Univariate and multivariate Cox proportional hazard analysis for progression-free survival in patients harboring epidermal growth factor receptor mutation and received tyrosine kinase inhibitors. [file 12885_2020_7765_MOESM2_ESM.docx]

Supplementary Table S2. Univariate and multivariate Cox proportional hazard analysis for progression-free survival in patients harboring epidermal growth factor receptor mutation and received tyrosine kinase inhibitors

|  | Univariate | | Multivariate | | | | | | |
| --- | --- | --- | --- | --- | --- | --- | --- | --- | --- |
|  |  | | Model-1 | |  | | Model-2 | | |
|  | HR (95% CI) | *p* value | HR (95% CI) | *p* value | |  | | HR (95% CI) | *p* value |
| Body mass index | 0.970 (0.938–1.003) | 0.072 | 0.963 (0.932–0.994) | 0.021 | |  | | 0.962 (0.932–0.994) | 0.020 |
| Smoking status | 1.171 (0.976–1.405) | 0.090 |  |  | |  | |  |  |
| Mutation types | 1.373 (1.142–1.650) | 0.001 | 2.806 (1.850–4.255) | <0.001 | |  | |  |  |
| Uncommon vs. classical | 2.147 (1.457–3.165) | <0.001 |  |  | |  | | 2.553 (1.715–3.802) | <0.001 |
| EGFR TKIs | 0.812 (0.703–0.938) | 0.005 |  |  | |  | |  |  |
| Afatinib vs. 1st generation TKIs | 0.722 (0.552–0.943) | 0.017 |  |  | |  | |  |  |
| Line of treatment | 1.474 (1.174–1.853) | 0.001 |  |  | |  | |  |  |

HR, hazard ratio; CI, confidence interval; EGFR, epidermal growth factor receptor; TKIs, tyrosine kinase inhibitors

The Model-1 included body mass index, smoking status, mutation types, epidermal growth factor receptor tyrosine kinase inhibitors, and line of treatment. The Model-2 included body mass index, smoking status, uncommon mutations versus classical mutations, epidermal growth factor receptor tyrosine kinase inhibitors, and ling of treatment.
