# Supplementary material for: Comparison of epidermal growth factor receptor tyrosine kinase inhibitors for patients with lung adenocarcinoma harboring different epidermal growth factor receptor mutation types
Source: BMC Cancer. 2021 Jan 11;21:52. doi: 10.1186/s12885-020-07765-6 (PMC7802134; doi:10.1186/s12885-020-07765-6)
Supplement: Supplementary file 3 — Additional file 3 Table S3. Drug-related serious adverse events. [file 12885_2020_7765_MOESM3_ESM.docx]

|  | Total | Afatinib | Erlotinib | Gefitinib | *p* value |
| --- | --- | --- | --- | --- | --- |
|  | n = 363 | n = 102 | n = 139 | n = 122 |  |
| Total | 27 (7.4) | 12 (11.8) | 8 (5.8) | 7 (5.7) | 0.145 |
| Skin | 18 (5.0) | 9 (8.6) | 5 (3.6) | 4 (3.3) | 0.105 |
| Gastrointestinal toxicity | 8 (2.2) | 3 (2.9) | 2 (1.4) | 3(2.5) | 0.715 |
| Myalgia | 1 (0.3) | 0 | 1 (0.7) | 0 | 0.446 |

Supplementary Table S3. Drug-related serious adverse events

Serious adverse events were defined as the grade 3 to 5 according to the National Cancer Institute Common Terminology Criteria for Adverse Events version 4.0
